# Autism Spectrum Disorder: Brain Areas Involved, Neurobiological Mechanisms, Diagnoses and Therapies

**DOI:** 10.3390/ijms25042423

**Published:** 2024-02-19

**Authors:** Jacopo Lamanna, Jacopo Meldolesi

**Affiliations:** 1 Center for Behavioral Neuroscience and Communication (BNC), 20132 Milan, Italy; lamanna.jacopo@hsr.it; 2 Faculty of Psychology, Vita-Salute San Raffaele University, 20132 Milan, Italy; 3 IRCCS San Raffaele Hospital, Vita-Salute San Raffaele University, 20132 Milan, Italy; 4 CNR Institute of Neuroscience, Milano-Bicocca University, 20854 Vedano al Lambro, Italy

**Keywords:** arborization, dendrite, dysgenesis, flat, haploinsufficiency, organoid, pre-/post-synapse, pre-school, spine, subtype

## Abstract

Autism spectrum disorder (ASD), affecting over 2% of the pre-school children population, includes an important fraction of the conditions accounting for the heterogeneity of autism. The disease was discovered 75 years ago, and the present review, based on critical evaluations of the recognized ASD studies from the beginning of 1990, has been further developed by the comparative analyses of the research and clinical reports, which have grown progressively in recent years up to late 2023. The tools necessary for the identification of the ASD disease and its related clinical pathologies are genetic and epigenetic mutations affected by the specific interaction with transcription factors and chromatin remodeling processes occurring within specific complexes of brain neurons. Most often, the ensuing effects induce the inhibition/excitation of synaptic structures sustained primarily, at dendritic fibers, by alterations of flat and spine response sites. These effects are relevant because synapses, established by specific interactions of neurons with glial cells, operate as early and key targets of ASD. The pathology of children is often suspected by parents and communities and then confirmed by ensuing experiences. The final diagnoses of children and mature patients are then completed by the combination of neuropsychological (cognitive) tests and electro-/magneto-encephalography studies developed in specialized centers. ASD comorbidities, induced by processes such as anxieties, depressions, hyperactivities, and sleep defects, interact with and reinforce other brain diseases, especially schizophrenia. Advanced therapies, prescribed to children and adult patients for the control of ASD symptoms and disease, are based on the combination of well-known brain drugs with classical tools of neurologic and psychiatric practice. Overall, this review reports and discusses the advanced knowledge about the biological and medical properties of ASD.

## 1. Generalities about ASD

Autism Spectrum Disorder, ASD, is a neurodevelopmental brain complex disease established in growing fetuses and very young children during early development. ASD is caused by the altered expression of specific genes [1,2]. Its identification was not an early discovery. In 1943, the comprehensive brain disease of origin, autism, had been recognized in very young, pre-school children affected by biomedical-to-psychological problems accompanied by problems of communication and social interaction [3,4,5]. During the following three decades, the study of autism in children continued. Their neurons were recognized to express chromosomal defects including perturbed genes revealed during their structure organization. In addition, ASD was found to be very complex especially from its pathogenic point of view. Moreover, its factors, involved in various effects, were very heterogeneous. In 1993, distinct components of autism disorder began to be proposed and then wishfully recognized. ASD appears as the most significant disease in the field. Initially, its definition was widely debated; now, it is generally accepted as the complex neurodevelopmental disorder established during, and caused by, genetic and environmental perturbations [1,2].

In ASD patients, however, the affected genes are not always the same. Rather, they are complicated by its extensive heterogeneity. In other words, each ASD gene is expressed by a fraction, neither by all affected patients nor by the recognized animal models [3,4,5]. This increased understanding made possible the inclusion in ASD of a number of subcategories, which is important for their distinct clinical and therapeutic properties [4]. However, all attempts made to identify various heterogeneous genes within clear subtypes and subcategories resulted in limited successes [4,6].

**Figure 1:**
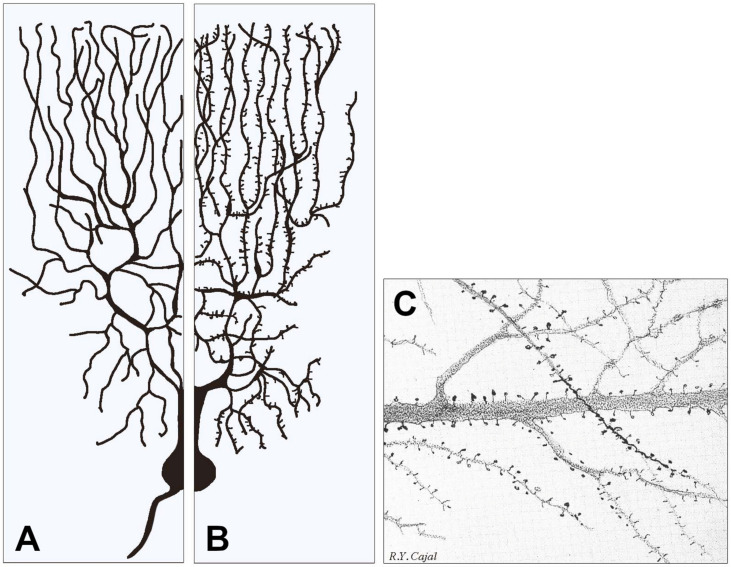
Examples of two types of dendrite arborization emerging from similar neuronal cell bodies and examples of dendritic spines. In (**A**), all dendritic fibers appear smooth because their post-synaptic structures, largely predominant in inhibitory neurons, are flat, i.e., they do not emerge or emerge only marginally from the fiber surface. In (**B**), the dendritic fibers predominant in stimulatory neurons are largely covered by spines, i.e., small stemming protrusions connected to fibers by their necks. (**A**,**B**) contain modified versions of Figure 1 from our previous publication [7]. (**C**) shows a fraction of an original figure by Santiago Ramon y Cajal (1896) (CAT 024, book Ciencia y Arte by the Instituto Cajal, Madrid, 2004) showing the heterogeneity of the spines emerging from dendritic fibers of pyramidal cells, illustrating in particular their variability in size, shape, density and distribution.

In the brain of children, the ASD distribution in the gray matter is wide, including structures such as the midbrain, pontine, bilateral hippocampus, left para-hippocampal gyrus, various temporal and occipital gyruses. Together with their affected genes, these structures sustain deficits of communication and restricted (or repetitive) behavior. In addition to their altered neurogenic effects, ASD was co-affected by abnormalities. Some properties of the latter, however, are still largely uncharacterized [1,2]. The principal targets of the disease are the synapses, in particular the post-synapses of dendrites, both flat and spiny (Figure 1). In addition to their general brain mentioned here, children with ASD show other, less frequent symptoms, including gastrointestinal dysfunctions and pulmonary hypertension. Organ alterations, such as scoliosis, are rare in children.

The child ASD frequency has been established in human populations. Up to 15 years ago, it was reported to account for 1.1% of the child population in the whole USA. As a consequence of recent intense investigations and advanced procedures employed, the calculated value corresponds to 2.3% of children. In a wider population, the affected children have been estimated to account for 1 out of 59 [1].

Given the extensive literature of ASD, it is vital to distinguish it from the other diseases. For this, in addition to the focus on child patients, it is important to illustrate various properties of the disease [8]. Therefore, the disease, including the aged form, has been defined also by psychodiagnostic tools in terms of DSM-5, i.e., according to the definition of Diagnostic and Statistical Manual for Mental Disorders from the American Psychiatric Association [9]. Prolonged treatments with factors have been analyzed also in mice [10]. By these definitions, we have emphasized the relevance of ASD in mental medicine, which is a concept that is re-discussed in the following Diagnosis Section 5.

The frequency of ASD in adult patients has been found to be close to the frequency in children just mentioned (2.2%) [1].

The present review is based on two main origins: established articles published one or two decades ago, reporting key documents about the existence and relevance of ASD, necessary for a recognized disease, followed by more recent publications including those that appeared during the last year. Within these two origins, many publications have been selected by direct comparison with others, which are similar in properties but less complete and precisely focused. Similar criteria have been used in the choice of ASD with respect to other subcategories, such as Asperger’s syndrome [11,12], which is also generated, but distinct, in the autism area. These other subcategories are not presented in parallel to ASD but rather only mentioned. General definitions such as intellectual disability (ID) and a few others, employed in previous publications for the completion of ASD properties [2], are not presented here in detail. In other words, our intention has been to inform our readers about ASD, including knowledge established recently.

## 2. The ASD Structures in the Brain

ASD children begin to be investigated during the first months of life, when the brain volume appears normal. During the subsequent months, the volume grows, reaching increases of approximately 10% at ages between 2 and 4 years. During late childhood and adolescence, the brain volume increases further but more slowly. Interestingly, the increased volume of many areas is more evident in the left than in the right brain. In particular, increases are reported in the hippocampal and temporal roles, in which however gray matter tends to decrease and white matter tends to increase [13]. Decreases of another structure, the cerebellum, are appreciable especially in adolescent and adult patients [14]. The details of structural properties, reported in many studies, have been investigated in populations of ASD patients. The considerable heterogeneity of the disease corresponds in many cases to functional alterations typical of various ASD subtypes [1,2,4,13,14].

Knowledge and understanding of the changes reported in ASD are critical to establish appropriately the state of neural structures in living patients. Adequate results for this purpose have been obtained by magnetic resonance imaging (MRI), which was initially employed more than a decade ago [15]. Recently, MRI has been employed in its multimodal forms, which are appropriate for early clinical diagnoses and also for present perspectives of therapy [16,17,18].

The changes of brain properties reported in ASD patients are largely due to their size and distribution. In children, the brain volume changes are primarily due to the increased surface area rather than cortical thickness [18,19]. White matter development can occur [19]; however, in several areas, the alterations are mostly due to gray matter. In the amygdala and hippocampus, the changes of gray matter, associated with the severity of autistic symptoms and language, are often reduced [20,21]. Other changes of ASD’s local properties take place during patient aging. For example, cortical gyrification is increased in children but decreases rapidly upon the development of adolescence [22].

## 3. Mechanisms of ASD Gene Operations: Key Role of Synapses

Gene variants of ASD, together with their regulatory nuclear factors often called risks, induce marked changes in a wide range of biological processes. The identification of some gene mutants has confirmed the ASD risks acting through largely distinct molecular pathways. Thus, the genes of ASD and the ensuing epigenetics are finely modulated in their human genomic context [23,24]. With time, the association of gene variants with ASD risks has been investigated, and many positive cases have been reported [25,26,27]. In some cases, however, the relevance of such variants has remained unclear. In order to solve the problems, some questions have been characterized in terms of nucleotide polymorphisms. The results have demonstrated the relevance of some genes and not other genes related to ASD [28]. Analogously unidentified ASD genes govern neuronal structure–function relationships in the cortex and other brain areas [29]. Moreover, a gene mutant, Sparcl1, and its protein Hevin, active as an ASD risk, have been found to induce, in the endoplasmic reticulum of neurons, a sort of stress characterized by structural instability [30]. The growing architecture induced by genetic variants is now providing innovative information about the pathological role of human ASD [26,31].

In addition to genes, two other factors are essential for ASD function: transcription factors (for example [32]) and the protein complexes active on chromatin remodeling. A very active subunit of the latter type, reported first in 2016 [33], is ARID1B. The latter complex, by interacting with various genes, plays a primary role in the growth of neurons and thus in ASD of children and adolescents [34]. Its knock-down was found to induce a decreased arborization of dendrites with an ensuing decrease in both excitatory and inhibitory intercellular communications [33,35].

Additional studies confirmed that marked changes of gene expression, operative with ARID1B, take place together with the decreased body size of the brain. The basic alterations include cortical inhibitory/excitatory imbalance with decreased GABAergic neurons and their transmission [35]. Interestingly, haploinsufficient mice are protected by the administration of growth hormone and also by early postnatal serotonin modulation [36]. ARID1B, therefore, appears to play an essential role in forebrain neurogenesis, inducing a pronounced role in inhibitory neural progenitors [34,35,36], with refinement of the ensuing progressive therapy [37]. In addition, available evidence has demonstrated that the role of ARID1B is not unique. Analogous effects are in fact induced by other chromatin remodeling subunits. This has been the case of KANSL1, WDR5 and a few others, which have been integrated into the present knowledge of ASD pathogenesis [2].

**Figure 2:**
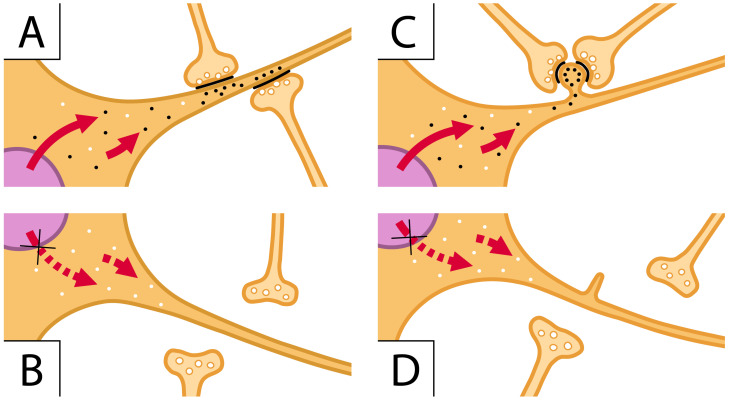
ARID1B acts as a scaffolding protein that holds together the components of its complex ability to operate with specific chromatin components of reactive genes. (**A**,**C**) images illustrate two cells (orange) characterized by flat and spiny dendritic fibers, respectively. In the nucleus (violet) of these images, the ARID1B complex regulates the transcription of specific genes. The generated mRNA transcripts (small black dots) are transferred to the cytoplasm of the corresponding proteins addressed to the dendritic fibers (red arrows). In (**A**), the latter proteins contribute to the appropriate assembly of flat post-synaptic structures. The bottom (**B**) is analogous to (**A**) except that ARID1B has been knocked-down, the red pointed arrows do not move specific mRNAs, small white dots contain proteins different from those generated by ARID1B, the post-synapses are absent, and the pre-synapses are scattered in the space. (**C**) is like (**A**) except for one spine with black dots assembled close to two pre-synapses assembling whole synapses; (**D**) corresponds to (**C**) without ARID1B; thus, it is analogous to (**B**) with respect to (**A**). The change in (**D**) versus (**C**) is the tiny spine to which pre-synapses assemble to establish the whole synapse.

Within neurons, the structures and fractions most widely affected by ASD genes and proteins correspond to, and interact with, various types and components of synapses [38]. In addition to intellectual and social effects, disabilities and speech defects, the impairment mutations of chromatin remodeling induced by ARID1B regulate primarily the dendritic differentiation in the developing brain [37]. The dysfunction of dendrites, the post-synaptic fibers that receive the pre-synaptic inputs, are critical for synaptic function, and they are also relevant in sensory processing, cognition and conscious perception [38]. The knock-down of ARID1B results in a block of stimulatory synaptic transmission (Figure 2).

Moreover, the integration of pre-synapses with dendritic excitability is reduced together with the number and morphology of dendritic spines. The dendrites with their post-synaptic responses to flat and spines are therefore sites of ASD gene effects [2,33,36,38]. In addition to the molecular complexes active as chromatin modifiers [36,37,38], ARID1B and its analogous subunits have been shown to govern other genes, encoding proteins localized at or near synapses [37,38,39] (Figure 2).

Specific aspects of synapses affected by ASD action need to be considered in structural and functional terms. In ASD pre-synapses, the structure and release of neurosecretory generation are less frequently affected [35,36], except for small boutons and glutamatergic synapses, whose post-synaptic mGluR is governed by Shank3 mutations and the interaction with neuroligin 1 [38,39,40]. On the other hand, the main structures of post-synapses, dendrites with their flat and spine structures, predominant in inhibitory and stimulatory synapses, respectively, are critical also because of their key role in the transmission from pre-synaptic structures [35,40]. The spines, tiny post-synaptic protrusions from dendrites that receive most of the excitatory synaptic input, are almost always affected by their role in ASD (Figure 1 and Figure 2) [2,36,40,41,42]. The predominant role of spines has been confirmed by studies where various aspects of synaptic ASD are investigated in parallel [37,38]. Functional and structural changes of spines are critical for synaptic plasticity, which is a cellular model of learning and memory. Altered spine morphology and plasticity are common markers of human neurodevelopmental disorders, such as conformational fluctuations and ID [43,44,45,46].

A final problem, not yet discussed in this review, deals with additional processes that activate ASD functions. In a mouse model, a dramatic increase in NO level induces a marked worsening of the ASD state. Moreover, high levels of nitrosative stress biomarkers and NO synthase inhibitors were found to induce changes of behavioral ASD-associated phenotype [47]. Analogous increases of oxidative stress were found to induce a polymorphism of the NRF2 gene, which is a master regulator of antioxidant stress [48]. Additional known stresses were found effective in the expression of ASD genes [49,50]. Interesting results were also obtained with human pluripotent stem cells assembled in organoid models [24]. These models, generated through the use of human pluripotent stem cells, produce profoundly intricate systems with spatiotemporal modeling of the developing brain by approaches that appear promising; however, they are still limited [51]. Based on previous suggestions and the present models, we can conclude that in the near future, some innovative ASD aspects will be recognized and characterized.

## 4. Contribution of Glial Cells

Up to now, the presentation of ASD and its regulation have been considered only of a neuronal nature. High numbers of glial cells, however, are present in the brain and at least two of them, astrocytes and microglia, discussed extensively in the literature during the last few years, participate in critical aspects of ASD. Here, we summarize a few relevant aspects of the glial cell role in ASD. Results of the two types of cells are reported, first concerning their separate effects [52,53,54,55,56] and then working together with coordinate effects [57,58].

In a normal brain, the number of astrocytes is considerable. In ASD-positive brains, such a number is decreased; however, the remaining astrocytes are often active. It can be concluded that astrocytes play a protective role on neuronal functions [53] with ensuing changes of synapse function [53]. The astrocyte roles, including brain inflammation, participate directly in the functions sustained by neurons [53,54].

The ASD role of the second type of glial cells Is focused more precisely. Microglia is strongly active in inflammation. Therefore, it does increase the process substantially. In numerous processes sustained by astrocytes together with neurons, microglia is inhibited. This occurs with neuroligin-4, which is a factor that interacts directly with ASD and operates also on other processes [55]. A factor that prevents inflammation is minocycline, which operates by modulating microglia polarization and therefore protecting ASD [56].

During the coordinate activations of the two glial cell types, some of their combined changes are relevant also for ASD. For example, microglia activation often induces astrocyte reactive activation and the ensuing release of ATP, which further activates microglia [57]. Moreover, chemokines and factors released by microglia are blocked by other factors released by astrocytes. The effects of both glial cells investigated in terms of ASD are shown in [58].

The most interesting interactions of glial cells include those with neurons. Specifically, accurate analyses of neuroinflammation, interactive with immunometabolic factors of glial origin, as well as immune mediators of ASD patients interactive with gene mutations, were reported to result in stress-positive responses [59]. The interest in these results was related to future therapies against immune abnormalities of children ASD [23]. On the other hand, ASD generation by growing fetuses was found to be reinforced by maternal antibodies; however, there was no convergence of stress functions [60]. Another important process, the generation of ASD markers, was shown to depend on miRNA expression by a serotonin transporter gene [61].

## 5. Diagnoses

ASD symptoms and pathological properties have already been mentioned previously. The goal of this section is not a repetition but rather the order presentation of advanced processes of diagnosis in relation to ASD alone and in combination/relation with other diseases. Diagnosing requires understanding how autistic patients react in response to various value-based paradigms [62,63]. This information should be considered in the early identification and diagnosis of ASD. At present, most family parents are concerned about their diseased children even before they turn 2 years old, even when their diagnoses are not made until age 4 or later. Under these conditions, the state of patients is often impaired due to their aggravating symptoms. Relevant especially for poor communities, recognition of the disease by experienced clinicians is essential in many terms: medical, operational and even economical [64,65]. For ASD children, diagnoses are often made by the application of advanced tests, such as the psychodiagnostic tests, a gold standard in diagnosing ASD, by including in the evaluations also the criteria of DSM-5 defined by the Diagnostic and Statistical Manual for Mental Disorders [9,66]. In case the results remain uncertain, additional distinct tests can be employed based on the experience of specialized centers [62,67].

In addition to children, analogous diagnostic efforts are made for adult ASD patients. For these symptoms, the efforts are numerous, including impaired social interactions, limited communication skills, and repetitive behaviors [9,63]. In addition, biochemical work-up can include body fluid analyses to reveal general metabolic and lysosomal storage properties; changes in autistic symptoms can result in self-injurious behaviors and psychomotor responses; genomic technology can be employed to identify molecular defects [26,27,68,69]. For patients of advanced age, appropriate diagnoses are very important also in term of destiny. Compared to the general population, in fact, their mortality is 2.9-fold higher [68].

Additional diagnoses of ASD are made based on specific properties we have already reported in previous sections. ARID1B haploinsufficiency is the well-known subunit complex effect of chromatin remodeling already presented [33,34,35,36]. Its related disorders, important for the recognition of ASD phenotypes, are highly heterogeneous. Animal models of this pathology have helped to identify the ASD molecular mechanisms where ARID1B participates in brain development [70]. In addition to molecular mechanisms, disease aspects are regulated by metabolic and cytoplasmic structures [27]. Convergent mechanisms underlying the dysgenesis of dendritic spines contribute to the distinction of ASD from other pathologies, which has been investigated in several animal models and in human post-mortem brain samples. Another concept developed by the study of ASD children, either alone or combined with another defect, is attention deficit hyperactive disorder (ADHD). The study confirmed ASD children expressing levels of anxiety higher than those of their peers [71]. They also exhibit higher levels when co-expressed with ADHD. Interestingly, results analogous to those of anxiety, induced by separate and combined ASD and ADHD, have been found also when ASD is combined with other processes such as gender dysphoria and impaired locomotor skills. Therefore, the results with child ASD together with ADHD can be interpreted as a form of psychiatric comorbidity [71,72].

Another example of ASD comorbidity is that of child sleep. The results demonstrated that sleep problems affect more than 95% of the patients and over 86% of their father parents. The latter 86% parents have more anxiety and depression than the parents of ASD children with no sleep problems. These results reveal how child sleep problems affect the well-being of parents [73]. Future research will establish whether comorbidities similar to those of sleep problems induce in parents also other effects, such as those induced in children by ASD [73].

The brain diseases and properties considered so far have been shown to depend directly on ASD. In other words, independent diseases co-occur with autistic defects. This state does not cover the whole types of ASD dependence. In the USA, when compared with the corresponding peers, the brain diseases expressed by ASD in children neurons induce depression frequencies of about 3-fold, anxiety of 2-fold, and epilepsy of over 20-fold [1]. Examples of corresponding therapy are reported in the following Section 7. Analogous studies have been made in adult ASD patients for neurodegenerative diseases assayed in terms of genes, symptoms and effects involved. An overlap of ASD risk genes has been found with schizophrenia. With neurodegenerative diseases, in particular with Parkinson’s and Alzheimer’s diseases, some gene commonality has emerged [74,75]. At present, however, details remain to be confirmed.

## 6. Therapies

The goal of many ASD studies, in particular those about diagnosis (see for example [59,69,74,75]), has been dedicated to specific therapies, first based on the present knowledge and then translated into clinical use. A problem of this approach is the heterogeneity of analyzed patients, especially of small infants, which is particularly difficult to characterize. The solution of this problem is expected from learning machines associated with electroencephalography and/or magnetoencephalography [76].

Drug employment is the mainstay of treatment for the core symptoms of ASD, including communication deficits, social interaction deficits and repetitive behavior. For the last 10 years, risperidone and aripiprazole, officially two antipsychotic drugs, are the only ones approved by the FDA to treat ASD children’s irritability. At present, the two drugs are employed in more than 30 countries. The doses per patient used in the USA and Europe are much higher than those in Turkey and other eastern countries [1,77]. However, the effectiveness of these drugs is limited, and adverse effects are frequent. The attempts of the industry for the production of new drugs or the employment of drugs already on the market are intense; however, the results are limited. Effects of cannabinoids and interest for probiotics, prebiotics and symbiotics are promising [78,79]; however, these are not yet approved.

At present, the ongoing ASD therapy is limited to well-known brain drugs prescribed to both children and adult patients for many ASD co-symptoms: anxiety, depression, hyperactivity, and sleep problems [80,81]. Among these drugs, those effective against attention-deficit/hyperactivity disorders are employed as psychotropic and psychostimulants [1,80,81,82]. Ongoing research, now intensely investigated, supports an association between ASD and immune/inflammatory mechanisms and proposes the development and future employment for specific inhibitory drugs [83]. Improvements of cognitive rehabilitation have been obtained by changes in the eye movement’s performance [84]. The administration of some such drugs induces various tolerability effects, starting by weight increase. Most often, the negative effects are controlled by adjustment of the employed doses [80,81,82,83].

Disorders not strictly dependent on ASD, such as gastrointestinal disorders, are also employed for ongoing therapy. Recent studies have revealed the brain mechanisms of such drugs. The microbiota gut–brain axis operates as a modulator of neuropsychiatric health. Many important functions, such as brain cognitive actions, as well as immunities, are governed by gut metabolites [85]. In combination with drugs, treatments include non-pharmacological interventions, such as behavioral therapy and acupuncture. Behavioral therapies, largely provided by doctors to both children and adult patients, have been often evaluated favorably based on high or moderate evidence [5,86]. Acupuncture has been found to stimulate some social functioning; however, the positive evidence of this therapy is limited [86]. Summing up, many well-known brain drugs are employed in ASD therapy with appreciable but not innovative results. A recent review has reconsidered critically the list of most such drugs. The results have largely confirmed the previous data. In addition, two long-term drugs, amitriptyline and loxapine, have been promising, deserving specific trials not yet dedicated to them for ASD diseases [87].

## 7. Conclusions

As specified in the title, the main interest of this review is focusing on various aspects of ASD medicine investigated upon the identification of ASD during the last few years. In contrast, we have left out the pre-medical properties of patients, critical for the discovery of autism in 1943, concerning the deficits of language, communication and social interactions. Here, we do not provide specific presentations of the latter processes. In case of interest by readers, they can be found in various forms of the literature such as [88,89].

Upon the identification of ASD, specific studies have identified many but not all of the properties, such as those of synapses. The study of these properties will be continued during the next few years. We expect ensuing developments, especially from the dendrites and their post-synaptic structures, and then the involvement of various medical brain specialties: from biomedicine to neurology, psychiatry, and also brain surgery. At the moment, also in non-advanced countries, the cooperation of these specialties is sustaining social and political initiatives.

A highly important development reported in this review concerns the expected progress of ASD therapy. At present, together with a few of marginal relevance, the drugs employed for ASD are the same employed for the other brain diseases. Therefore, innovative drugs are needed, and the knowledge appears promising for future developments [90,91]. The emergence of pharmaceutical industrial initiatives appears to be even more innovative. If the numbers of appropriate polymers exposed to manufacturing technologies are limited, ongoing work is expected to develop new ASD drug formulations [92].

## Abbreviations

ADHD	attention deficit of hyperactive disorder
ARID1B	subunit of a protein complex active in chromatin remodeling
ASD	autism spectrum disorder
ID	intellectual disability
MRI	magnetic resonance imaging
