# Correction: Lamanna, J.; Meldolesi, J. Autism Spectrum Disorder: Brain Areas Involved, Neurobiological Mechanisms, Diagnoses and Therapies. *Int. J. Mol. Sci.* 2024, *25*, 2423

**DOI:** 10.3390/ijms27052233

**Published:** 2026-02-27

**Authors:** Jacopo Lamanna, Jacopo Meldolesi

**Affiliations:** 1Center for Behavioral Neuroscience and Communication (BNC), 20132 Milan, Italy; 2Faculty of Psychology, Vita-Salute San Raffaele University, 20132 Milan, Italy; 3IRCCS San Raffaele Hospital, Vita-Salute San Raffaele University, 20132 Milan, Italy; 4CNR Institute of Neuroscience, Milano-Bicocca University, 20854 Vedano al Lambro, Italy

## Text Correction

There was an error in the original publication [1]. A correction has been made to the section “1. Generalities about ASD” by removing the paragraph starting with the sentence “Consequences of aging are structural alterations often resulting from the progressive degenerations […]”. The paragraph was removed because it included comments on studies that were improperly selected due to acronym confusion and do not relate to autism spectrum disorder (references [11–16]).

In addition, the acronym ASD (autism spectrum disorder) was improperly typed as “ADS” in a few instances: sections “1. Generalities about ASD”, “5. Diagnoses”, “7. Conclusions”.

## References

The acronym ASD was corrected in the title of reference [8]:

8.Maurice, V.; Russet, F.; Scocco, P.; McNicholas, F.; Santosh, P.; Sing, S.P.; Street, C.; Purper-Ouakil, D. Transition from child and adolescent mental health care to adult services for young people with attention-deficit/hyperactivity disorder (ADHD) or autism spectrum disorder (ASD) in Europe: Barriers and recommendations. *Encephale* **2022**, *48*, 555–559.

Furthermore, with this correction, the order of references from 17 onwards has been adjusted accordingly due to removal of the following references [11–16]:

11.Ohyama, S.; Kotani, T.; Iijima, Y.; Okuwaki, S.; Sunami, T.; Iwata, S.; Sakuma, T.; Ogata, Y.; Akazawa, T.; Shiga, Y.; et al. Incidence and potential risk factors of superior mesenteric artery syndrome after spinal corrective surgery in patients with adult spinal deformity. *World Neurosurg.* **2023**, *5*, e591–e598.12.Mir, J.M.; Galetta, M.S.; Tretiakov, P.; Dave, P.; Lafage, V.; Lafage, R.; Schoenfeld, A.J.; Passies, P.G. Achievement and maintenance of optimal alignment following adult spinal deformity corrective surgery: A 5 years outcome analysis. *World Neurosurg.*
**2023**, *180*, e523–e527.13.Lee, B.J.; Bae, S.S.; Choi, H.Y.; Park, J.H.; Hyun, S.J.; Jo, D.J.; Cho, Y. Proximal junctional kyphosis or failure after adult spinal deformity surgery-review of risk factor and its prevention. *Neurospine* **2023**, *20*, 863–875.14.Dalton, J.; Mohammed, A.; Akioyamen, N.; Schwab, F.J.; Lafage, V. Preoperative planning for adult spinal deformity goals: Level selection and alignment goals. *Neurosurg. Clin. N. Am.* **2023**, *34*, 527–536.15.Elsayed, Y.M.H.; Almarghany, A.A. Resolution of trifascicular heart block with effective closure of congenital atrial septal defect followed by later coronavirus disease 2019. *J. Innov. Card. Rhythm. Manag.* **2023**, *14*, 5533–5536.16.Kawabata, A.; Sakai, K.; Yamada, K.; Utagawa, K.; Hashimoto, J.; Morishita, S.; Matsukura, Y.; Oyaizu, T.; Hirai, T.; Inose, H.; et al. The lower osteotomy level is associated with decreased *revision* surgery due to mechanical complications after tree column osteotomy in patients with adult spinal deformity. *Glob. Spine J.* **2023**, *18*, 21925682231196449.

The authors state that the scientific conclusions are unaffected. This correction was approved by the Academic Editor. The original publication has also been updated.
